# Echoviruses are a major cause of aseptic meningitis in infants and young children in Kuwait

**DOI:** 10.1186/1743-422X-7-236

**Published:** 2010-09-16

**Authors:** Ajmal Dalwai, Suhail Ahmad, Widad Al-Nakib

**Affiliations:** 1 Department of Microbiology, Faculty of Medicine, Kuwait University, Kuwait

## Abstract

**Background:**

The etiologic agents of aseptic meningitis (AM) often include human enteroviruses. The role of enteroviruses causing AM in young children was investigated during a 3-year period in Kuwait.

**Results:**

Enteroviral RNA was detected in cerebrospinal fluid (CSF) by reverse transcription-PCR and specific genotypes of enteroviruses were identified by direct DNA sequencing of VP4-VP2 region. Enteroviral RNA was detected in 92 of 387 (24%) suspected AM cases and the results were confirmed by hybridization of amplicons with an internal, enterovirus-specific probe. The CSF samples from 75 of 281 (27%) children < 2 years old but only from 3 of 38 (8%) 4-12 year-old children were positive for enteroviral RNA (*p *= 0.011). Majority of infections in children < 2 years old (49 of 75, 65%) were due to three echoviruses; echovirus type 9 (E9), E11 and E30. Only three other enteroviruses, namely coxsackievirus type B4, coxsackievirus type B5 and enterovirus 71 were detected among AM cases in Kuwait.

**Conclusions:**

Our data show that three types of echoviruses (E9, E11 and E30) are associated with the majority of AM cases in Kuwait. To the best of our knowledge, this is the first report to characterize different enterovirus genotypes associated with AM in the Arabian Gulf region.

## Introduction

Aseptic meningitis (AM) is a severe, potentially fatal infection of the central nervous system (CNS) and is characterized by meningeal inflammation that is not associated with any identifiable bacterial pathogen in the cerebrospinal fluid (CSF) [1]. Most patients with AM present with abrupt onset of fever accompanied by complaints of headache, stiff neck, lethargy, anorexia, and may also experience vomiting, diarrhea, sore throat and rash. The CNS involvement in neonates may not be accompanied by overt signs of meningeal inflammation. The AM is frequently caused by viral agents, particularly the human enteroviruses (EVs) belonging to the family *Picornaviridae *[1,2]. More than 10,000 cases of AM are reported annually to The Centers for Disease Control and Prevention. Children are more susceptible than adults to infections by these viruses [3]. The CNS disease in newborns caused by EVs may also progress to meningoencephalitis with the appearance of seizures and focal neurological deficits [1,2].

The EVs are small, nonenveloped, single stranded RNA viruses that are transmitted mainly through fecal oral route and can cause sporadic cases, outbreaks and epidemics [4]. The EVs have been classified into 68 distinct serotypes and new enteroviruses are being described based on molecular characterization [5,6]. The capsids of EVs are made up of four structural proteins (VP1 to VP4) of which VP1 as well as VP4-VP2 region sequences have been used for typing human EVs [7,8]. In the U. S., some serotypes such as coxsackievirus type B5 (CB5), echovirus 6 (E6), E9 and E30 were associated with epidemics and outbreaks in various years during 2003-2005 while others such as coxsackievirus type A9 (CA9), CB3 and CB4 and enterovirus 71 (EV-71) were endemic throughout the period [9]. During 1998 to 2001, EV-71 was associated with several outbreaks in Taiwan with hand, foot and mouth disease and severe encephalitis while more recently several outbreaks of AM due to E30 have been reported [6,9-12]. Most studies have shown that predominant strains of enteroviruses change over time at a given location and majority of infections are seen during summer to fall season [1,2,11,13]. On the contrary, a persistence of AM cases due to EVs in winter months was preceded by a large outbreak in spring and summer in France in 1999 [14]. Although AM usually has a benign course and treatment options are limited, surveillance of AM due to EVs is crucial for early identification of such cases to avoid further testing, inappropriate use of antimicrobials and to arrest intrafamilial spread of EV infection [2,15]. There is no information on the role of EVs causing AM in Kuwait or other adjoining countries in the Arabian Gulf region of the Middle East. This 3-year study was carried out to determine the role and type of EVs causing AM cases in Kuwait, an Arabian Gulf country in the Middle East, and the results obtained are reported here.

## Results

### Demographic and clinical data

During the three-year period of this study, CSF samples from 387 suspected AM patients were collected and investigated for enteroviral RNA. Of these, 281 (73%), 68 (18%) and 38 (10%) samples were obtained from children < 2 years of age, 2-4 years old and 4-12 year old, respectively. The number and percentage of patients in different age groups presenting with symptoms suggestive of AM are shown in Table 1. Symptoms of fever, flu-like illness, poor appetite and signs of meningeal inflammation were apparent in vast majority of children (Table 1).

**Table 1:** Clinical data for < 2 year old (n = 281), 2-4 year old (n = 68) and 4-12 year old (n = 38) children who presented with symptoms of aseptic meningitis

Disease symptoms	No. (%) of subjects presenting with symptoms in		
	
	< 2 year old	2-4 year old	4-12 year old
Fever	281 (100)	68 (100)	38 (100)

Flu-like symptoms	257 (91)	65 (96)	38 (100)

Poor appetite	275 (98)	45 (66)	33 (87)

Photophobia	N.D.	54 (79)	27 (71)

Disturbed consciousness	125 (44)	12 (18)	0 (0)

Rash	136 (48)	11 (16)	2 (5)

Signs of meningeal inflammation	227 (81)	35 (51)	32 (84)

N.D., not determined

### Detection of enteroviral RNA by RT-PCR and probe hybridization

Overall, enteroviral RNA was detected in 92 of 387 (24%) patients by single step RT-PCR (Table 2). All RT-PCR-positive amplicons also yielded a positive hybridization signal with an internal enterovirus-specific probe, as expected. The positivity for enteroviral RNA in CSF samples varied among children of different age groups and was significantly lower in older children. Thus, 75 of 281 (27%) CSF samples from children < 2 years old were positive for enteroviral RNA while only 3 of 38 (8%) samples from older (4-12 years) children were positive for enteroviral RNA (*p *= 0.011). The positivity for enteroviral RNA in CSF samples from children 2-4 years old was also higher compared to older children (14 of 68, 21% Vs. 3 of 38, 8%; *p = 0.088*) but slightly lower than in children < 2 years of age (14 of 68, 21% Vs. 75 of 281, 27%; *p = 0.3*), but the differences were not statistically significant (Table 2). Consistent with earlier reports [1,2], two distinct peaks of enteroviral activity were also noted in Kuwait with majority of enteroviral infections occurring during summer (May-June) and fall (Nov.-Dec.) months in each year of the three-year study period (data not shown).

**Table 2:** Positivity for enteroviral RNA in cerebrospinal fluid (CSF) samples from suspected aseptic meningitis cases among children of various age groups in Kuwait

Age of subjects	Total no. of CSF samples analyzed	No. of CSF samples positive for enteroviral RNA	% Positivity
< 2 years	281	75*	27

2-4 years	68	14	21

4-12 years	38	3*	8

All subjects	387	92	24

*Difference statistically significant (*p *= 0.011)

### Genotype classification of enteroviruses by DNA sequencing of VP4-VP2 region

Based on VP4-VP2 region sequencing, only six genotypes were detected and their occurrence was sporadic indicating that no outbreaks of enteroviral AM occurred during the three-year period in Kuwait. The robustness of VP4-VP2-based genotypic classification was checked by virus isolation and antibody neutralization test from sixteen selected enterovirus-positive CSF samples. In each case, the serotype identified by virus neutralization test was identical to the genotypic classification based on VP4-VP2 sequencing (data not shown). The VP4-VP2 region sequences of different enteroviruses from Kuwait exhibited 92% to 97% identity with the prototype strains of various specific enteroviruses. Majority (60 of 92, 65%) of enteroviral AM cases in Kuwait were caused by three types of echoviruses, E9, E11 and E30 (Table 3). The E9 and E11 alone accounted for the majority (43 of 75, 57%) of infections while EV-71 and CB5 were the other two common genotypes causing AM in children < 2 years of age. Interestingly, 97% (29 of 30) of E9 infections and 93% (14 of 15) of EV-71 infections were seen among children < 2 yrs old (Table 3). The different genotypes in AM cases in older children were more evenly distributed (Table 3).

**Table 3:** Distribution of specific enterovirus genotypes among enterovirus-positive cerebrospinal fluid samples of suspected aseptic meningitis cases from children of various age groups in Kuwait

Age of subjects	No. of enterovirus positive samples	No. (%) of specific enterovirus genotypes identified					
		
		CB4*	CB5	E9	E11	E30	EV-71
< 2 years	75	1 (1)	11 (15)	29 (39)	14 (19)	6 (8)	14 (19)

2-4 years	14	2 (14)	2 (14)	1 (7)	5 (36)	3 (21)	1 (7)

4-12 years	3	0 (0)	1 (33)	0 (0)	2 (67)	0 (0)	0 (0)

All subjects	92	3 (3)	14 (15)	30 (33)	21 (23)	9 (10)	15 (16)

*CB4: coxsackievirus type B4; CB5: coxsackievirus type B5; E9, echovirus type 9; E11: echovirus type 11; E30, echovirus type 30; EV-71: enterovirus 71

## Discussion

This three-year study investigated the role of EVs in suspected AM cases in Kuwait. The enteroviral RNA was detected by a sensitive, one-step RT-PCR assay that could detect as few as 50 copies of enteroviral genome [16] and the reproducibility of the assay was periodically checked by both internal as well as external National External Quality Assurance Testing (UK-NEQAS) with consistent performance. This is important since ~34% of various in-house RT-PCR assays developed for enterovirus detection have been reported to fail quality testing [17]. The enteroviral meningitis cases by different EVs were scattered throughout the period of the study and no outbreaks were observed. Furthermore, all major enterovirus genotypes were detected every year except E30 that appeared during the third year of the study. The frequency of enteroviral AM in 92 of 387 (24%) infants and young children in Kuwait is comparable with annual surveillance data from countries such as USA, UK and some other countries [9,14,18,19] but slightly lower than that reported from other countries such as Spain and Canada [20,21]. Data from the Middle East is generally lacking. Few reports based on virus isolation procedures have found a much lower frequency of enterovirus meningitis in Jordan and Tunisia [22,23]. On the contrary, much higher frequency of enterovirus meningitis has been observed in outbreaks caused by different enteroviruses [6,10,12,24].

The majority (75 of 92, 82%) of the enteroviral meningitis cases were among children < 2 yrs of age. Furthermore, the frequency of AM cases in children < 2 yrs of age (75 of 281, 27%) was significantly higher than the frequency (3 of 38, 8%) seen in older (4-12 year) children (*p *= 0.011). The findings are consistent with earlier observations showing that infants and young children, due to their developing immune system, are more susceptible to enteroviral infections [1,13]. Enteroviral infections in older children are less common and are often associated with recreational water activities [2].

The VP1 region has been mostly used for genotypic classification of enteroviruses since antibody neutralizing epitopes are generally located on surface exposed loops of this protein, however, other investigators have also successfully used VP4-VP2 region for genotyping of enteroviruses [8,25]. We also used sequencing of VP4-VP2 region and the genotypic classification based on this approach for a selected panel of enterovirus-positive samples was completely concordant with the serotype obtained by virus neutralization test. A recent study has also shown complete concordance between enterovirus genotypes determined by sequencing of VP1 region with those derived from VP4 and VP2 regions [26]. Only six genotypes of EVs were detected with nucleotide sequence identities ranging from 92% to 97% over the entire length of the ~650-bp amplicons with the prototype strains of different enteroviruses. The data are consistent with reports that only few dominant genotypes are endemic causing sporadic AM cases in a given geographical setting [1,2]. The three echoviruses (E9, E11 and E30) caused majority (60 of 92, 65%) of AM cases in Kuwait with E9 being most common. Echoviruses, particularly E6, E9 and E30 have also been most commonly found in both, sporadic and outbreak AM cases in USA, Canada and several other countries [2,9,11,21]. The E30 which has recently been associated with outbreaks in several countries was less common while E6 was not even detected in Kuwait. The lower frequency of E30 and absence of E6 infections could be related to the absence of any outbreaks of AM in Kuwait during the study period. The detection of E11 as the second most common etiologic agent of enteroviral meningitis was rather surprising and may be related to its higher occurrence in the Middle-Eastern region. The detection of EV-71, mostly found in Southeast and East Asian countries, in younger children is most likely due to the presence of a large number of expatriate population originating from this region that works as domestic helpers in Kuwait [27,28].

A limitation of the present study is the large number (76%) of AM cases that did not have an enteroviral etiology. It is probable that some of the enterovirus-negative samples were due to lack of extraction of viral RNA or presence of PCR inhibitors in nucleic acid recovered from these clinical samples since no internal control was used with each sample. Other possible explanations for the lower positivity may include delay in reporting to the hospital after the onset of AM as the initial symptoms of fever and vomiting commonly seen in younger children are often regarded as routine. Furthermore, these cases are usually referred to the hospital by peripheral polyclinics where the patients first report. Studies have shown that the higher positivity of RT-PCR is obtained if CSF samples are collected at an early stage after the onsent of AM, typically within two days, due to declining viral load [29]. It has also been shown that many of the RT-PCR positive specimens remain virus culture negative due to lower viral load. A recent study from Japan showed that virus culture-positive CSF samples contained significantly higher echovirus genome copy numbers than did virus culture-negative CSF samples and E9 was the predominant echovirus identified [30]. Since E9 has also been found to attain higher viral load in CSF specimens in Kuwait [16], it is probable that its increased detection in AM cases is related to the higher load of this virus in CSF samples than other EVs. It is also probable that at least some of the cases were classified as AM due to prior treatment with antibiotics, non-infectious etiologies as a result of certain drug ingestions (such as amoxicillin- or ibuprofen- or trimethoprim-sulfamethoxazole-induced AM) or due to other viral agents that were not explored in this study [1,2]. To the best of our knowledge, this is the first report providing data on the prevalence and association of specific enterovirus genotypes with AM in the Arabian Gulf region.

## Materials and methods

### Patient's data

This study prospectively identified 387 children < 12 years of age who were admitted from September 2003 to August 2006 to the paediatric units of two major (Mubarak Al-Kabir and Adan) hospitals in Kuwait with the clinical diagnosis of AM. All children underwent lumbar puncture for collection of cerebrospinal fluid (CSF) which was routinely cultured for common bacterial pathogens in the hospital microbiology laboratory and a portion of CSF sample was transported to the Reference Virology Laboratory, Faculty of Medicine, Kuwait University where RT-PCR tests and virus culture for enteroviruses were performed. The study was approved by the Ethics Committee of the Faculty of Medicine, Kuwait University. Demographic, laboratory and clinical data were collected. Laboratory findings included CSF pleocytosis and absence of bacterial growth. A case was defined as AM based on the following criteria: fever (>38°C), symptoms and signs of meningeal inflammation, CSF white blood cell count >5 × 10^6^cells/l and negative CSF bacterial culture.

### Viral RNA isolation

The viral RNA was isolated from the CSF samples, typically within one working day, using the viral RNA isolation kit (Qiagen) according to manufacturer's instructions and as described previously [16]. For the detection of enteroviral RNA by reverse transcription-PCR (RT-PCR), 1 μl of extracted RNA was used.

### Detection of enteroviral RNA

Enteroviral RNA was detected in RNA samples by using the RT-PCR kit (Qiagen) and a sensitive, one-step RT-PCR protocol described previously [16]. The samples were analyzed by agarose gel electrophoresis, as described previously [31]. The reproducibility of the assay was periodically checked by both internal as well as external National External Quality Assurance Testing (UK-NEQAS) with consistent performance.

### Southern blotting and hybridization

The enterovirus-specific identity of amplicons was confirmed by Southern hybridization using an enterovirus-specific biotin-labeled probe, EVKP (5'-biotin-ATTGTCACCATAAGCAGCCA-3' corresponding to nucleotide positions 601 to 582 of prototype E9 strain DM, GenBank accession number AF524867). The amplicons, after agarose gel electrophoresis, were transferred onto positively charged nylon membranes and the hybrids were detected by using anti-biotin antibodies as described previously [16,31].

### Viral isolation in cell culture

The virus was isolated from some selected samples by using African green monkey kidney (Vero) and HeLa cells cultured in a monolayer on 24-well microplates. The cells were cultured for at least two weeks at 37°C and were periodically checked for characteristic cytopathogenic effect. If a cytopathogenic effect was observed, the sample was considered as virus-culture positive. The isolated virus was subsequently identified by virus neutralization test using neutralization antiserum specific to each enterovirus.

### Genotyping of enteroviruses by DNA sequencing

A DNA fragment (~650 bp) covering the hypervariable VP4-VP2 region [8] of enteroviral genome was amplified from each enterovirus-positive CSF sample by using the one-step RT-PCR protocol described above except that primers EVK1 (5'-GACTACTTTGGGTGTCCGTGT-3' corresponding to nucleotide positions 543 to 563 of prototype E9 strain DM, GenBank accession number AF524867) and EVK2 (5'-GGTAAYTTCCACCACCANCC-3' corresponding to nucleotide positions 1197 to 1178 of prototype E9 strain DM, GenBank accession number AF524867) were used. A portion (5 μl) of the RT-PCR product was run on agarose gel to confirm the amplification of a DNA fragment of expected size and the remaining sample was purified by using QIAquick PCR purification columns (Qiagen) as described previously [32,33]. Both strands of purified amplicons were sequenced by using cycle DNA sequencing kit (DTCS CEQ8000, Beckman Coulter) as described in detail previously [32,33] except that primer EVK1 or EVK2 were used as sequencing primers. Reverse complements were generated and aligned with forward sequences using ClustalW http://www.ebi.ac.uk/Tools/clustalw/index.html. GenBank identity search using Basic Local Alignment Search Tool (BLAST) http://www.ncbi.nlm.nih.gov/BLAST/Blast.cgi? was performed for genotype identification. A minimum identity of 92% over the entire length of the amplicons with prototype strains of different enteroviruses was used for genotype assignment [8]. The DNA sequencing data reported in this study have been submitted to EMBL under accession numbers FR687401 to FR687410 and FR690863 to FR690867.

### Statistical analyses

Differences in proportions were compared by using χ*^2 ^*test. A *P *value of < 0.05 was considered as statistically significant. All statistical analyses were performed using WinPepi software ver. 3.8 (PEPI-for Windows).

## List of Abbreviations

AM: aseptic meningitis; CNS: central nervous system; EV: enteroviruses; E9: echovirus 9; E11: echovirus 11; E30: echovirus 30; CB4: coxsackievirus B4; CB5: coxsackievirus B5; EV-71: enterovirus 71; VP; viral capsid protein.

## Competing interests

The authors declare that they have no competing interests.

## Authors' contributions

SA and WAN designed the study. AD performed the experiments and analyzed the data. All authors contributed in writing and approved the final manuscript.

## Author Details

Virology Unit, Department of Microbiology, Faculty of Medicine, Kuwait University P.O. Box 24923, Safat 13110, Kuwait
